# The potent potential of MFAP2 in prognosis and immunotherapy of triple-negative breast cancer

**DOI:** 10.1007/s12672-024-01044-7

**Published:** 2024-06-01

**Authors:** Jing Huang, Yuting Xu, Shengnan Qi, Qi Zheng, Can Cui, Lei Liu, Fan Liu

**Affiliations:** 1 Department of Oncology, Affiliated Hospital of Nantong University, 20 Xisi Road, Chongchuan District, Nantong, 226001 China; 2 Department of Pathology, Affiliated Hospital of Nantong University, 20 Xisi Road, Chongchuan District, Nantong, 226001 China; 3 The First Clinical Medical College of Nanjing Medical University, Nanjing, 211166 China; 4 Department of Pathology, Qingdao Eighth People’s Hospital, Qingdao, 266121 China

**Keywords:** MFAP2, Triple-negative breast cancer (TNBC), Immunotherapy, Tumor mutational burden (TMB), PD-L1

## Abstract

**Backgrounds:**

Microfibril-associated protein 2 (MFAP2) is a protein presenting in the extracellular matrix that governs the activity of microfibrils through its interaction with fibrillin. While the involvement of MFAP2 in metabolic disorders has been documented, its expression and prognostic significance in triple-negative breast cancer (TNBC) remain unexplored.

**Methods:**

We acquired datasets pertaining to breast cancer (BC) from the Gene Expression Omnibus (GEO) and The Cancer Genome Atlas (TCGA) databases. Next, a Venn diagram was used to identify the differentially expressed genes (DEGs). The DEGs were used to perform Gene Ontology (GO), Kyoto Encyclopedia of Genes and Genomes (KEGG), protein–protein interaction (PPI), immune and survival analysis. The expressions of MFAP2, PD-1 and PD-L1 were examined by immunohistochemistry and western blot and their relationship with clinical pathological parameters were analyzed by clinical specimen samples from patients with TNBC. Tumor Immune Estimation Resource (TIMER, https://cistrome.shinyapps.io/timer/) was adopted to calculate the immune infiltration level of TNBC. The link between gene expression and tumor mutational burden (TMB) was described using Spearman’s correlation analysis.

**Results:**

We identified 66 differentially expressed genes (DEGs) that were up-regulated. Among these DEGs, MFAP2 was found to be overexpressed in TNBC and was associated with a lower probability of survival. This finding was confirmed through the use of immunohistochemistry and western blot techniques. Additionally, MFAP2 was found to be related to various pathological parameters in TNBC patients. Mechanistically, gene set enrichment analysis (GSEA) revealed that MFAP2 primarily influenced cellular biological behavior in terms of epithelial mesenchymal transition, glycolysis, and apical junction. Notably, MFAP2 expression was positively correlated with the abundance of macrophages, while a negative correlation was observed with the abundance of B cells, CD4 + T cells, CD8 + T cells, neutrophils and dendritic cells through immune analysis. Furthermore, it was observed that MFAP2 displayed a negative correlation not only with tumor mutational burden (TMB), a recognized biomarker for PD-1/PD-L1 immunotherapy, but also with PD-L1 in samples of TNBC.

**Conclusion:**

MFAP2 may be an important prognostic biomarker for TNBC, as well as a viable target for immunotherapy in this disease.

**Supplementary Information:**

The online version contains supplementary material available at 10.1007/s12672-024-01044-7.

## Introduction

Breast cancer (BC) exhibits heterogeneity and can be categorized into two subtypes: triple-negative breast cancer (TNBC) and non-triple-negative breast cancer (NTNBC). TNBC is distinguishable from NTNBC by significant clinicopathological features such as a higher chance of recurrence, bigger tumor size, lymph node metastases, and a worse prognosis [1]. Approximately 15–20% of invasive BC cases are identified as TNBCs. TNBC is identified by the lack of progesterone receptors, estrogen receptors, and HER2 expression [2]. In comparison to other forms of BC, triple-negative breast cancer exhibits an earlier onset and a greater degree of malignancy [3]. TNBC represents a subtype with unfavorable prognosis in contrast to other subtypes of BC due to the absence of efficacious treatment options [4]. Despite the extensive investigation into molecular alterations associated with TNBC, identifying effective immune-related biomarkers for TNBC is crucial for reducing mortality and developing innovative targeted therapies.

At present, systemic chemotherapy is still the recommended treatment for TNBC [5]. First-line chemotherapy drugs like taxane, anthracycline and platinum drugs are widely used to treat locally progressed TNBC patients. However, with a high proportion of recurrence and distant metastasis, the outcome of traditional chemotherapy remains unsatisfying [6]. Whereas, immunotherapy, which has been shown to increase patient survival in other solid cancers, is now giving a viable treatment option for TNBC [7]. Immune checkpoint inhibitors (ICIs), such as CTLA-4 and PD-1, are presently the most routinely utilized immunotherapeutic medicines. By blocking receptors such as CTLA-4 and PD-1, they can improve the cytotoxicity and proliferation potential of tumor-infiltrating lymphocytes (TILs). It is reported that TNBC is more likely to benefit from immunotherapy, because there are more tumor-infiltrating lymphocytes in TNBC tissues, which means TNBC may respond to immunotherapy [8, 9]. Moreover, TNBC usually exhibited high frequency of nonsynonymous mutations, which leads to neoantigen-specific T cells activated by high expression of tumor-specific neoantigens and thus the anti-tumor immune response was initiated [10, 11].

As the field of bioinformatics has advanced, an increasing number of novel biomarkers have been developed to assist in the identification and prognosis of cancer [12, 13]. In our study, we utilized a bioinformatic approach to identify 66 up-regulated differentially expressed genes (DEGs) from four datasets of triple-negative breast cancer. Among these DEGs, MFAP2, or microfibril-associated glycoprotein 1, a component of extracellular elastic microfibrils, was the gene of interest.

Multiple studies have confirmed the association between MFAP2 mutation and various conditions in humans, including hemostasis and thrombosis, metabolic disease, thoracic aneurysms, and osteopenia [14–16]. An inquiry exploring the potential link between MFAP2 expression and several forms of malignant cancer has recently been conducted. According to Li et al. MFAP2 is upregulated in gastric cancer cells and increases motility via the MFAP2/integrin α5β1/FAK/ERK pathway [17]. Furthermore, it has been proposed that MFAP2 might improve the transition between epithelial and mesenchymal in gastric carcinoma via activating the TGF-β/SMAD2/3 pathway [18]. Limited research has been conducted on the involvement of MFAP2 in TNBC.

This study employed a combination of bioinformatic methodology and clinical samples to ascertain MFAP2 as a prognostic biomarker exclusive to TNBC. The investigation encompassed an examination of MFAP2 expression, its protein interaction networks, and enrichment pathways to elucidate potential mechanisms. Furthermore, the relationship between MFAP2 expression and clinical pathological indicators was investigated in order to verify its prognostic importance. Additionally, the association of MFAP2 expression with immune infiltration, as well as immune checkpoints (PD-1/PD-L1), was examined to predict the efficacy of immunotherapy. The findings of this study might imply that MFAP2 is an essential prognostic marker for TNBC as well as a possible therapeutic target.

## Materials and methods

### Human tissue samples

The cohort for this study consisted of 301 BC samples and normal samples, obtained from the Department of Pathology at the Affiliated Hospital of Nantong University between February 2014 and December 2021. The fresh tissues of TNBC were collected between June 2021 and October 2021. Prior to participation, all patients were provided with comprehensive information and signed written informed consent to participate and publish. This study followed the standards established by the Helsinki Declaration and was approved by the Affiliated Hospital of Nantong University's ethics committee.

### Data collection and processing

The Cancer Genome Atlas (TCGA) and Gene Expression Omnibus (GEO) databases are widely utilized repositories of tumor-related data. We obtained the original datasets from both the TCGA and GEO databases. In our study, a total of four datasets were acquired, three from GEO and one from TCGA. To account for potential false-positive findings, an adjusted P-value was computed for the GEO datasets. Differential expression of messenger RNAs (mRNAs) was determined by applying a threshold of adjusted P < 0.05 and Log2 (Fold Change) > 1 or Log2 (Fold Change) <  − 1.

### Screening DEGs

The GEO database (http://www.ncbi.nih.gov/geo) was utilized to obtain datasets related to BC. Additionally, the TCGA database (https://portal.gdc.com) was used for collecting RNA sequencing profiles and accompanying clinical information for both BC and noncancerous samples. The raw data in MINiML files was downloaded and analyzed in R using the Limma package to identify genes with differential expression. Subsequently, the datasets were further analyzed using Venn diagrams to select the files containing DEGs. Integration of genes from the GEO and TCGA databases was performed.

### Analyzing DEGs for functional annotation and pathway enrichment

Functional enrichment analysis was utilized to figure out the underlying functionalities of the potential targets. Gene Ontology (GO) was utilized as a tool for annotating genes based on various ontologies, encompassing biological processes (BP), cellular components (CC), and molecular functions (MF). The Kyoto Encyclopedia of Genes and Genomes (KEGG) serves as a sophisticated database resource that facilitates the systematic examination of gene functions by establishing connections between genomic information and high-order functional data. In order to gain a deeper comprehension of mRNA carcinogenesis, we utilized the ClusterProfiler package (version: 3.18.0) in the R programming language to conduct an analysis of the Gene Ontology function and the KEGG pathway of the targets. The generation of the heatmap was accomplished through the utilization of the Pheatmap package in R.

### Construction of a PPI network

The STRING online tool was employed to assess and integrate information pertaining to protein–protein interactions (PPIs), including their physical and functional associations. In order to further evaluate the interactions and functions of the DEGs, we utilized the STRING and Cytoscape platforms to construct a PPI network.

### Survival analysis of MFAP2

We utilized the Kaplan–Meier plotter tool to validate the predictive effectiveness of our target gene. Specifically, we investigated the relationship between MFAP2 and overall survival (OS) in individuals with TNBC. To confirm MFAP2's predictive effectiveness, we used a Cox proportional hazards regression analysis for univariate and multivariate analyses of prognostic values. P < 0.05 was considered to indicate a statistically significant difference. Furthermore, we carried out an analysis of MFAP2 expression in different subtypes of BC using UALCAN (http://ualcan.path.uab.edu/index.html). Time-dependent ROC analysis was conducted using R version 4.0.3's pROC packages to compare prognostic values for OS.

### Gene set enrichment analysis (GSEA)

We downloaded the TCGA-BRCA geneset from the TCGA platform and divided it into two groups based on the expression levels of MFAP2. GSEA was employed to identify potential functions for MFAP2. To calculate normalized enrichment scores for each analysis, 1000 gene set permutations were performed. The cut-off condition was considered for adjusted P < 0.05.

### Immune infiltration, and tumor mutational burden (TMB) analysis

We examined the relationship between MFAP2 and immune infiltration using the "Gene" module of Tumor Immune Estimation Resource (TIMER, https://cistrome.shinyapps.io/timer/). The connection between gene expression and TMB was calculated using Spearman's correlation analysis.

### Immune checkpoint analysis of TNBC

In the dataset of the TCGA, RNA-sequencing expression profiles as well as clinical information about TNBC are provided (https://portal.gdc.com). A total of eight immune-checkpoint–relevant transcripts were selected for analysis: SIGLEC15, TIGIT, PD-L1, HAVCR2, PD-1, CTLA4, LAG3 and PDCD1LG2.

The algorithms and R packages described above were written in R foundation for statistical computing (2020) version 4.0.3. The R packages Ggplot2 and Pheatmap were used.

### Western blot analysis

We extracted proteins from fresh tumors and adjacent tissues of TNBC patients using lysis buffer containing protease inhibitors. For collection of the supernatant, the mixture was centrifuged for 20 min at 12,000 rpm at 4 °C. After boiling for 15 min at 100 °C with 5 × sodium dodecyl sulphate loading buffer, protein samples were obtained. In 12% sodium dodecyl sulfate polyacrylamide gel electrophoresis (SDS-PAGE), proteins were separated and transferred to polyvinyl difluoride membranes (Millipore, USA). MFAP2 and ACTB primary antibodies were incubated at 4 °C for 24 h after membranes were blocked for 2 h with 5% skimmed milk. An enhanced chemiluminescent kit (Millipore, USA) was used to visualize target bands after washing with TBST three times and incubating with specific antibodies for two hours. Finally, the quantification of protein bands was completed using Image J.

### Immunohistochemistry analysis

Antigens were extracted using a high-pressure pretreatment with EDTA buffer (pH 9.0). Endogenous peroxidase was then eliminated with 3% hydrogen peroxide, followed by avidin blocking for 20 min. The slides were incubated overnight at 4 °C with rabbit polyclonal antibodies against MFAP2 (1:2000, Abcam, USA, ab231344), PD-1 (ZSGB-BIO, China, ZM-0381) and PD-L1 (1:500, Abcam, USA, ab228415), followed by 30 min at room temperature with secondary antibodies. Tissue slices were counterstained and dehydrated after a 10-min incubation with 3,3-diaminobenzidine. Independent assessment and evaluation of immunohistochemistry analysis results was conducted by two expert pathologists. Based on the staining intensity, we had four evaluation criteria: negative (0), weak (1), medium (2), and strong (3). The staining extent was calculated as the proportion of positively stained spots in relation to the whole cancer area and was graded as 0, 0%, 1, 1–25%, 2, 26–50%, 3, 51–75%, and 4, 76–100%. The combination of the extent and intensity scores was used as the final expressed results, which were rated as follows: −, score 0–2; + , score 3 or 4; +  + , score 5 or 6; or +  +  + , score 7. Here, − and 1 + represent low expression; and 2 + and 3 + represent high expression.

### Statistical analysis

R (version 4.0.3 and version 4.1.3) and SPSSPRO software (version 1.0.11) were used to analyze the data. To examine survival outcomes, Kaplan–Meier curves were utilized. Wilcoxon test was performed to assess statistical difference of two groups and Kruskal–Wallis test was utilized to compare significance difference of more than three groups. Chi-squared test was employed to examine the relationship between MFAP2 expression and patient clinical status. The two-gene association was drawn using the R software package ggstatsplot. Spearman’s correlation was used to assess gene expression correlations. P-values of less than 0.05 were deemed statistically significant.

## Results

### DEG analysis

**Fig. 1:**
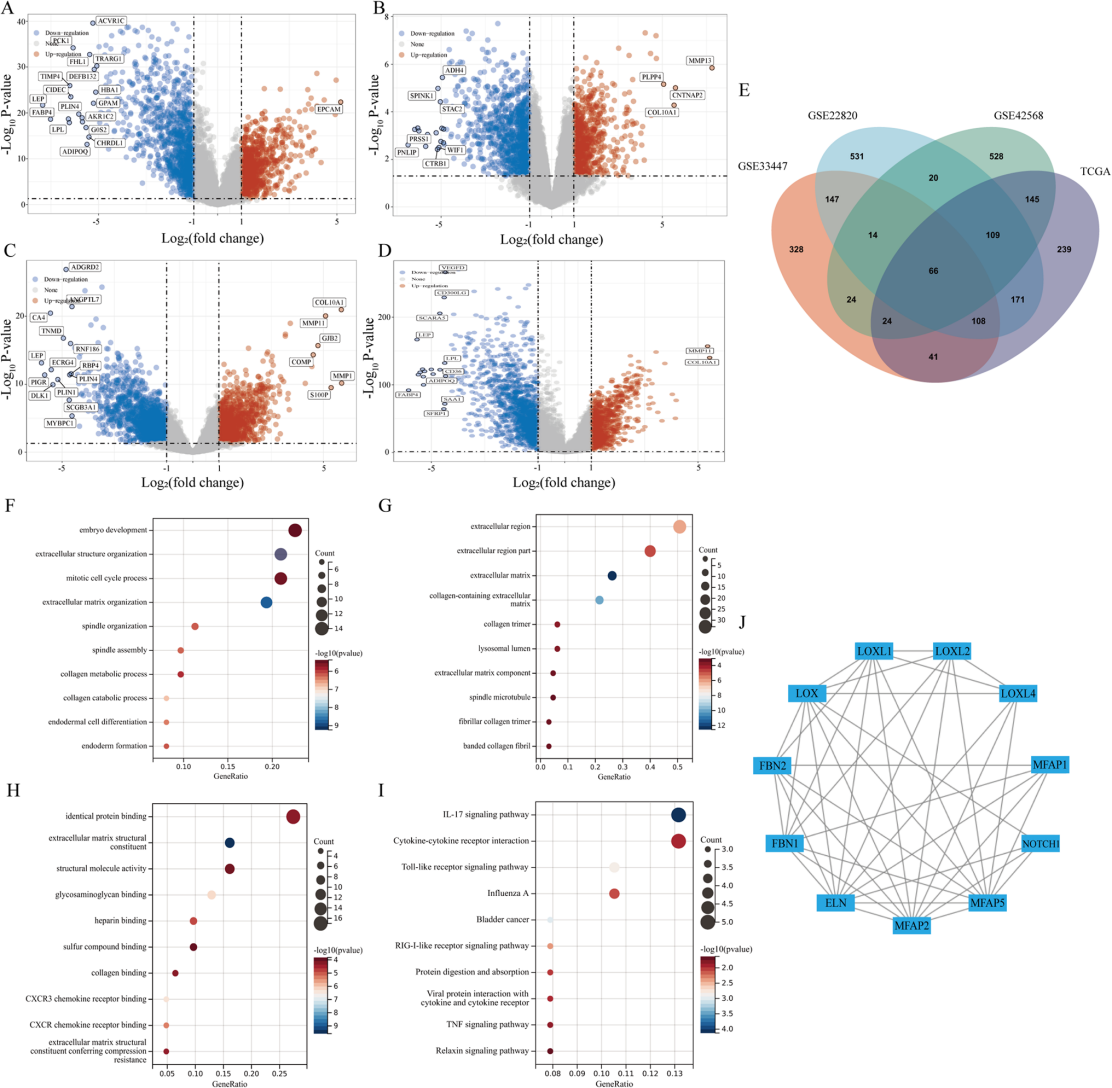
Identification and function analysis of DEGs. **A** Volcano plot of GSE42568. **B** Volcano plot of GSE33447. **C** Volcano plot of GSE22820. **D** Volcano plot of TCGA. **E** Venn plot of the up-regulated DEGs of the four datasets. **F** Bubble map of GO enrichment analysis in BP. **G** Bubble map of GO enrichment analysis in CC. **H** Bubble map of GO enrichment analysis in MF. **I** Bubble map of KEGG pathway analysis. **J** PPI network of MFAP2

From the GEO database, 288 BC samples and 35 normal samples were retrieved, specifically from the datasets GSE42568, GSE33447, and GSE22820. Additionally, the TCGA database was used to retrieve a dataset including 1101 BC samples and 113 normal samples. The DEGs from these datasets were analyzed using the Limma package (Fig. 1A–D, Tables S1–S4). Our study primarily focused on the up-regulated genes, resulting in the identification of 66 up-regulated DEGs from the four datasets using the Venn program (Fig. 1E, Table S5).

### Analyzing GO and KEGG and constructing a PPI network

The GO categories of the up-regulated DEGs were examined to ascertain the functional categories in which the DEGs were implicated. The findings of the GO analysis demonstrated that, in terms of Biological Processes (BP), the DEGs exhibited significant enrichment in embryo development, extracellular structure organization, mitotic cell cycle process, and extracellular matrix organization. Furthermore, in the Cellular Component (CC) analysis, the DEGs were predominantly associated with the extracellular region, extracellular region part, and extracellular matrix. Furthermore, the Molecular Functions (MF) of identical protein binding, extracellular matrix structural constituent, and structural molecule activity were identified (Fig. 1F–H). To gain further insights into the up-regulated DEGs, a KEGG pathway analysis was conducted. The findings revealed that the DEGs were predominantly enriched in the IL-17 signaling pathway and cytokine-cytokine receptor interaction, followed by the toll-like receptor signaling pathway and Influenza A, as depicted in Fig. 1I. Among all the up-regulated DEGs, MFAP2 was ultimately selected as the target gene for our study. To better understand the molecular mechanisms that influence BC, the STRING database was used for establishing the PPI network (threshold = 0.9). Using Cytoscape's MCODE plugin, we identified ten genes whose expression products were highly connected to those of MFAP2 (Fig. 1J).

### Evaluation of the prognostic relevance of MFAP2 in BC

**Fig. 2:**
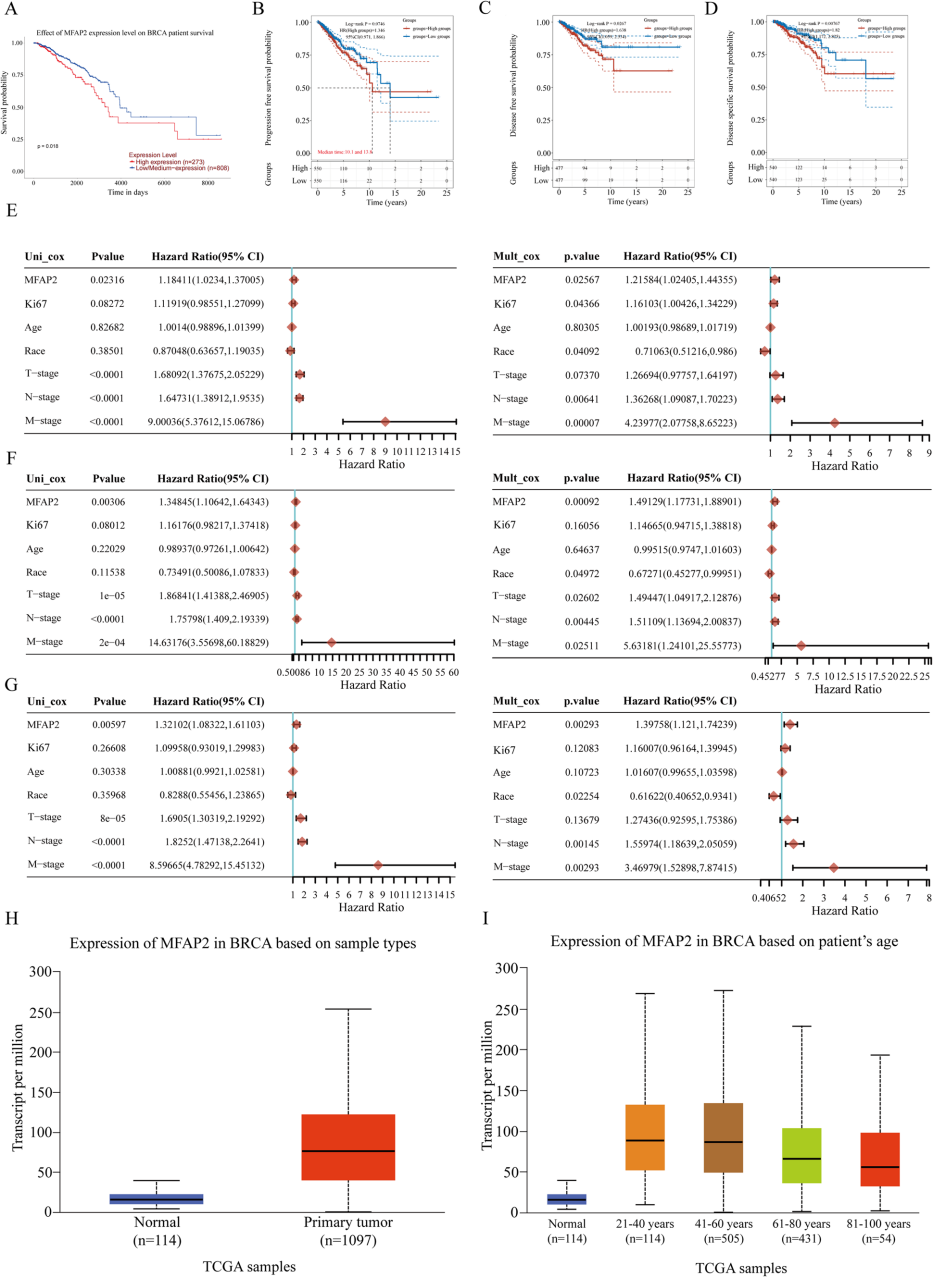
In patients with BC, high expression of MFAP2 was associated with poor survival. **A** Kaplan–Meier curves from the UALCAN were used to analyze the relationship between MFAP2 expression and OS in BC patients. **B**–**D** The relationship between MFAP2 expression and PFS/DFS/DSS in BC patients was analyzed using Kaplan–Meier curves derived from the TCGA datasets. **E** Univariate and multivariate cox regression analyses of PFS in patients with breast cancer. **F** Univariate and multivariate cox regression analyses of DFS in patients with breast cancer. **G** Univariate and multivariate cox regression analyses of DSS in patients with breast cancer. **H** Correlation between MFAP2 expression and sample types. **I** Correlation between MFAP2 expression and BC patient’s age

To validate MFAP2’s probable prognostic value, we used UALCAN to explore the relationship between MFAP2 levels and overall survival in patients with BC. The survival curves revealed a significant reduction in overall survival rate among patients who had elevated MFAP2 expression levels in comparison to individuals with reduced expression levels (P = 0.018). Furthermore, survival analysis conducted on data from TCGA yielded consistent findings, revealing a comparable association between MFAP2 expression level and progression-free survival (PFS), disease-free survival (DFS), and disease-specific survival (DSS) (Fig. 2A–D). To further explore the potential of MFAP2 in predicting the survival conditions of BC patients, we optimized univariate and multivariate cox regression analyses. Univariate analysis showed that high MFAP2 expression (P = 0.02316), T stage (P < 0.0001), N stage (P < 0.0001) and M stage (P < 0.0001) correlated with the PFS of BC patients. Then, we incorporated the variables into a multivariable cox regression analysis. The results demonstrated that MFAP2 (P = 0.02567), Ki67 (P = 0.04366), race (P = 0.04092), N stage (P = 0.00641) and M stage (P = 0.00007) were independent prognostic factors in BC (Fig. 2E). We also confirmed that MFAP2 was an independent prognostic factor for DFS and DSS of BC patients (Fig. 2F, G). An analysis of MFAP2 expression levels was performed utilizing the online tool UALCAN, which revealed that expression levels in BC tissues were greater than in tissues that were normal (Fig. 2H; P = 1.62E−12) and there was a correlation between its expression and patient’s age (Fig. 2I).

### MFAP2 expression was upregulated in TNBC tissues

**Fig. 3:**
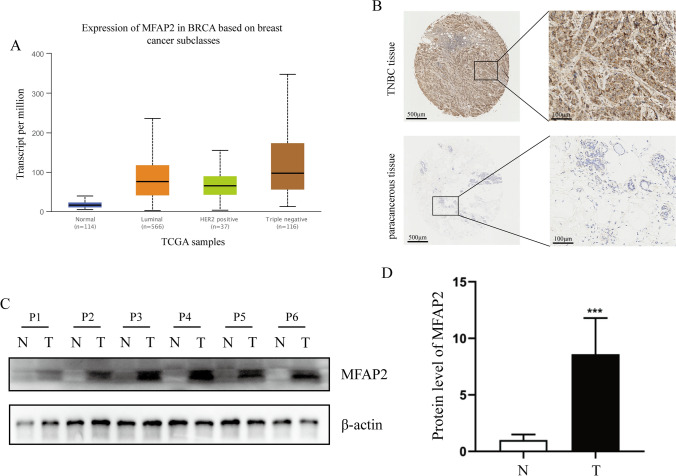
Detection and validation of MFAP2 expression in TNBC patients. **A** Correlation between MFAP2 expression and BC subtypes. **B** The immunohistochemistry results in our cohort. Magnification: 40 and 200. Scale bar: 500 μm and 100 μm. **C**, **D** The protein expression of MFAP2 in 6 pairs of TNBC tissues

During our study, a noteworthy correlation between MFAP2 expression and breast cancer subtypes was observed. The expression investigation demonstrated a substantial association between MFAP2 expression and BC subclasses (Luminal, HER2 Positive, and TNBC), with particularly high expression levels observed in TNBC. These findings suggested that MFAP2 had the potential to be utilized as a biomarker for diagnosing the TNBC subclass (Fig. 3A). To validate the expression and significance of MFAP2 in TNBC, immunohistochemistry was conducted on tumor tissues and surrounding tissues from 301 TNBC patients. The results showed that positive MFAP2 staining was stronger in tumors than in paracancerous tissue of TNBC patients (Fig. 3B). Furthermore, six pairs of samples from TNBC and paracancerous tissues were employed for western blot verification, and MFAP2 protein expression was clearly elevated in TNBC tissues (Fig. 3C, D). The results of immunohistochemistry and western blot demonstrated that MFAP2 expression level was up-regulated in TNBC tissues, suggesting MFAP2 plays a significant role in the development of TNBC. Thus, the above findings suggested that MFAP2 expression may be a predictor for unfavorable outcomes in BC, especially in TNBC patients.

### Validation of the prognostic value of MFAP2 in clinical data

**Fig. 4:**
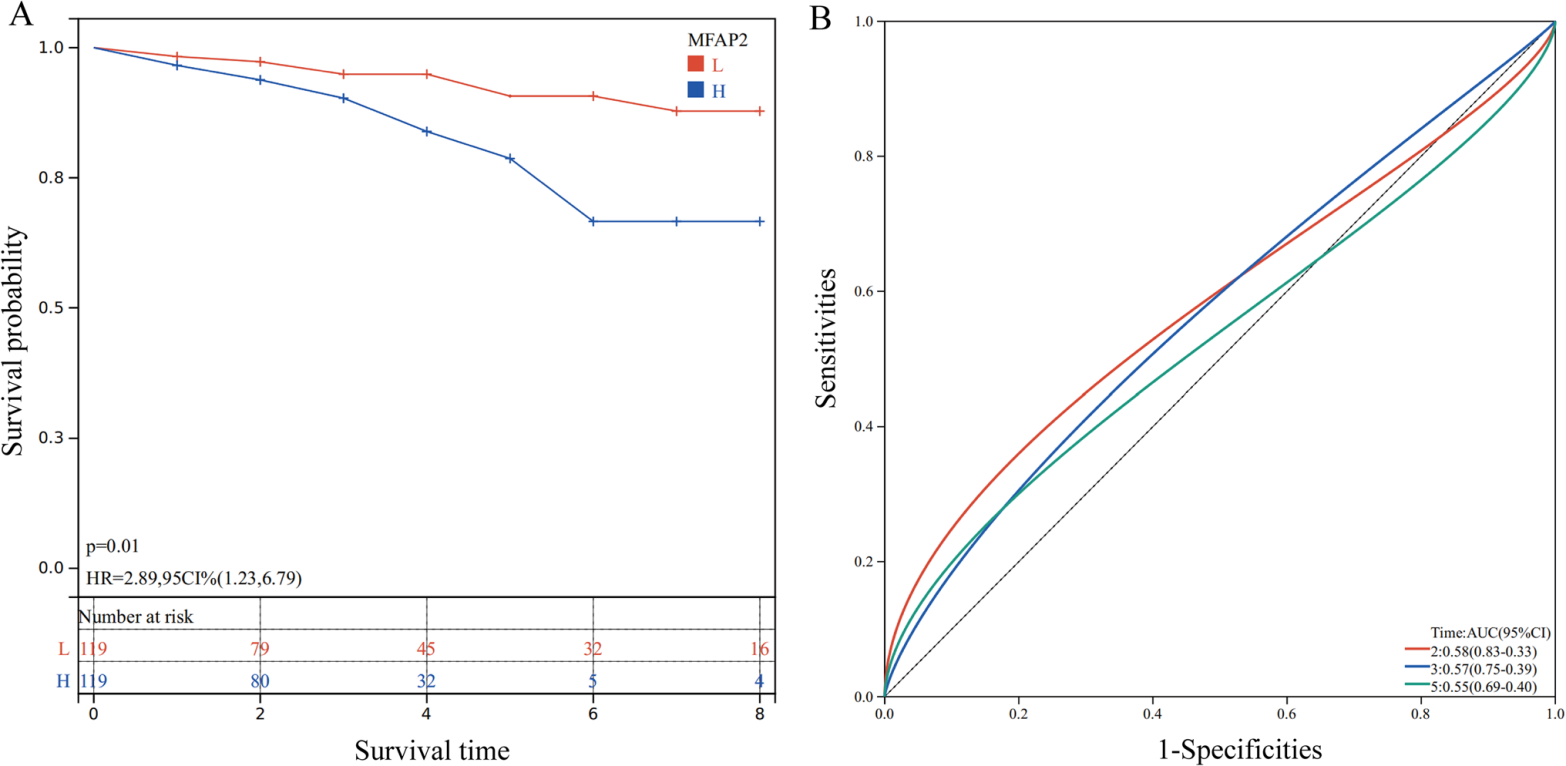
Prognostic value of MFAP2 in TNBC. (**A**) The relationship between MFAP2 expression and OS in clinical 238 TNBC patients was analyzed by Kaplan–Meier curves. (**B**) ROC analysis of MFAP2 expression in TNBC based on time

To confirm our previous hypothesis, we employed follow-up data obtained from 238 patients as a test cohort. High and low risk groups have been defined based on the degree of MFAP2 expression of the patients. According to the KM curve, MFAP2 had a predictive effect in TNBC, with patients in the relatively low-risk group having longer OS than those in the group with a higher risk (Fig. 4A). Moreover, the AUCs of 2-, 3- and 5- survival in the ROC curve were displayed in Fig. 4B. According to the results, patients with TNBC who expressed high levels of MFAP2 had a poor prognosis. In addition, we investigated the relationship between MFAP2 transcription and several clinical features, and our findings revealed that MFAP2 gene expression was related to ki67 expression (P = 0.007) and tumor grade (P = 0.030) (Table 1).

**Table 1:** Relationship between MFAP2 expression and pathological parameters of TNBC patients

Clinicopathological parameters	All cases	MFAP2 expression		P-value
		High	Low	
Age				
< 60	165	89	76	0.152
≥ 60	56	24	32	
Grade				
3	129	74	55	0.030**
2	63	30	33	
1	29	9	20	
Tumor diameter				
≤ 2 cm	91	45	46	0.908
2–5 cm	122	64	58	
> 5 cm	8	4	4	
Lymph node metastasis				
0	149	75	74	0.518
< 10	55	31	24	
≥ 10	17	7	10	
ki67				
> 35	171	94	77	0.007***
15–35	36	10	26	
≤ 15	14	9	5	
PD-L1				
L	145	64	81	0.004***
H	76	49	27	

*P<0.1; **P<0.05; ***P<0.01

### An analysis of the GSEA between groups with high and low expression of MFAP2

**Fig. 5:**
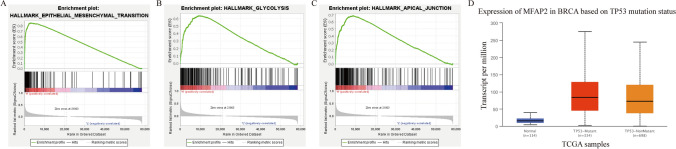
Comparison of groups with high and low expression of MFAP2 by GSEA. **A**–**C** The application of GSEA was utilized to detect enriched biological processes in samples exhibiting high expression of MFAP2. **D** The relationship between expression of MFAP2 and TP53 mutation status was revealed by analyzing the TCGA-BRCA database

In GSEA, we evaluated datasets involved in the highly expressed samples in the TCGA-BRCA dataset in order to determine how MFAP2 might function. In the TCGA database, a GSEA analysis was performed on two groups of patient samples based on their level of MFAP2 expression. The biological effects of MFAP2 on TNBC cells are mainly reflected in the epithelial-mesenchymal transition, glycolysis, and apical junction, as shown in Fig. 5A–C. Besides, TP53 mutation can facilitate tumor cell survival [19]. Therefore, we compared the expression level of MFAP2 expression in TP53-mutant and TP53-nonmutant patients. The results showed that BC patients with the TP53 mutation expressed considerably more MFAP2 than BC patients absent the mutation or normal people (Fig. 5D). In conclusion, MFAP2 may play an important role in tumorigenesis of BC.

### Correlation between MFAP2 and immune infiltration and its potential as a prognostic indicator for immunotherapy effectiveness

**Fig. 6:**
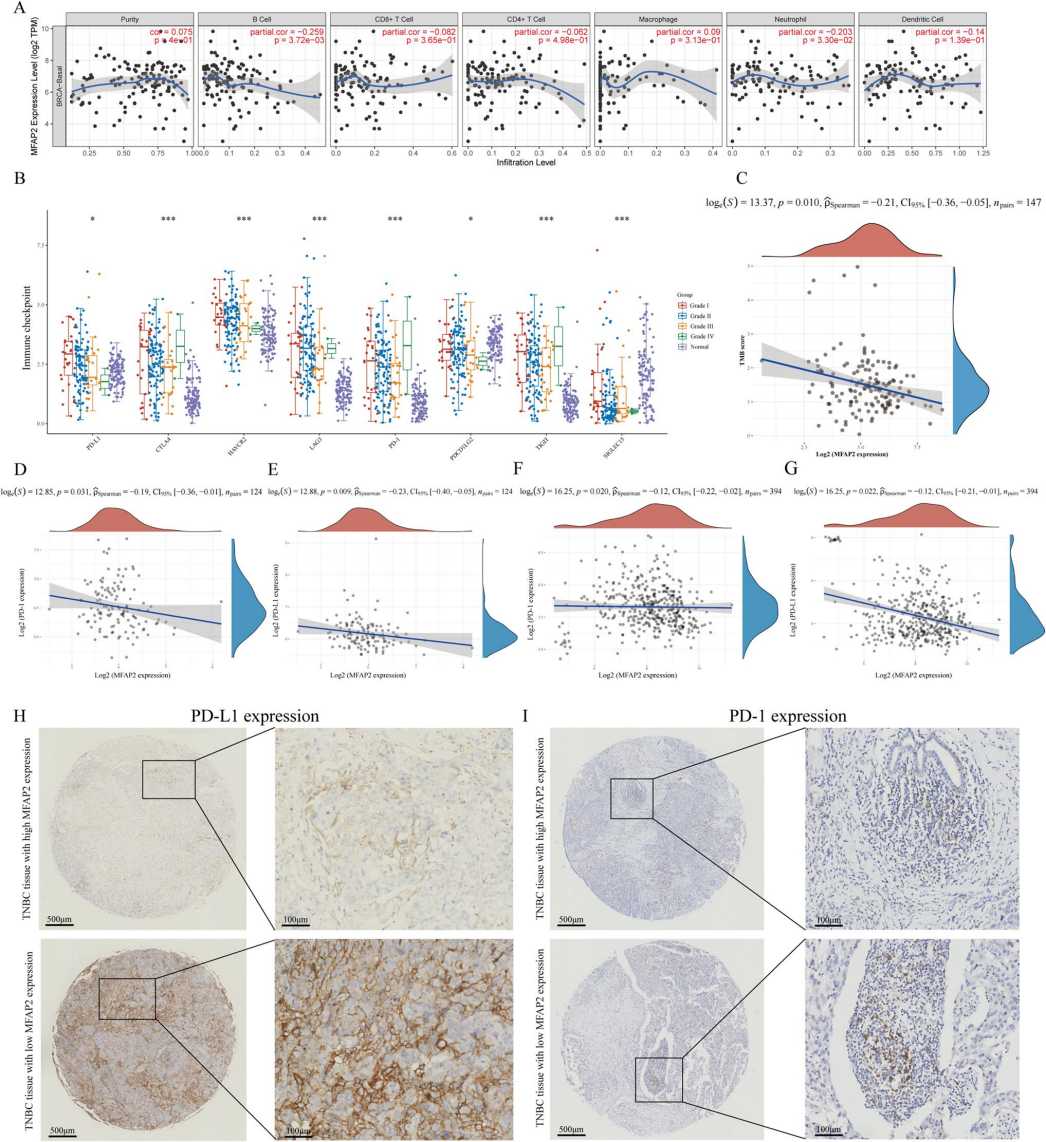
Immune infiltration analysis of MFAP2. **A** MFAP2 expression is associated with immune cell abundance. **B** Different expressions of immune-checkpoints in TNBC and normal tissues. **C** The correlation between MFAP2 and TMB in TNBC. **D**, **E** Correlation analysis between the expression of MFAP2 and PD-1/PD-L1 in GSE167213. **F**, **G** Correlation analysis between the expression of MFAP2 and PD-1/PD-L1 in GSE137356. **H** Immunohistochemistry results indicating low PD-L1 expression in patients with high MFAP2 expression and high PD-L1 expression in patients with low MFAP2 expression. **I** Immunohistochemistry on PD-1 in TNBC tissues with varied MFAP2 expression. Magnification: 40 and 200. Scale bar: 500 μm and 100 μm

The TIMER network analysis tool was used to study the link between MFAP2 transcription and immune infiltration in TNBC. We examined the connection between MFAP2 expression and six immunological cells (B cells, CD4 + T cells, CD8 + T cells, neutrophils, macrophages, and dendritic cells). We discovered a weakly significant connection between MFAP2 expression and tumor purity (r = 0.075, P < 0.05). However, macrophage abundance was positively correlated with MFAP2 expression, while other immune infiltrates were negatively correlated (Fig. 6A). According to these results, we analyzed the differential expressed immune-checkpoint genes SIGLEC15, CTLA4, PD-L1, HAVCR2, LAG3, PD-1, TIGIT and PDCD1LG2 in TNBC tissues of different grades and normal tissues. The findings illustrated that PD-L1, CTLA4, HAVCR2, LAG3, PD-1, and TIGIT expression were up-regulated in TNBC tissues. And the expression of PDCD1LG2 and TIGIT were down-regulated in TNBC tissues (Fig. 6B). These data suggested that MFAP2 might be potential immunotherapy targets.

TMB was employed as an emerging biomarker for TNBC immunotherapy. We studied the connection between MFAP2 transcription and TMB in TNBC. The findings demonstrated a negative relationship between TMB and MFAP2. These findings demonstrated that MFAP2 might be used as a prognostic indicator to predict immunotherapy response (Fig. 6C).

### Correlation of MFAP2 with PD-L1 expression in TNBC

Because we discovered a negative association between the levels of MFAP2 and TMB in the prior investigation and in the meantime, high TMB forecasts patients’ higher sensitivity to PD-1/PD-L1 immunotherapy, we supposed that MFAP2 expression and PD-L1 expression are correlated in TNBC. To confirm the hypothesis, we calculated the expression level of the genes in two GEO datasets (GSE167213 and GSE137356). The findings revealed that whereas MFAP2 expression increased, PD-L1 expression decreased significantly (Fig. 6D–G; P < 0.05). To further investigate, immunohistochemistry was used to assess the degree of PD-L1 expression in TNBC patients who had varied levels of MFAP2. As illustrated in Fig. 6H, patients with lower MFAP2 expression exhibited higher levels of PD-L1, and MFAP2 was related to PD-L1 expression (P = 0.004, Table 1), indicating a greater potential for these TNBC patients to benefit from immunotherapy. However, the immunohistochemistry on PD-1 in TNBC tissues didn’t reveal any significant correlation between the two molecules (Fig. 6I). Based on the aforementioned research, we have demonstrated the potential of MFAP2 to serve as a significant biomarker for immunotherapy, offering valuable guidance for clinical treatment of TNBC.

## Discussion

In this study, we examined MFAP2 expression and discovered that MFAP2 was especially up-regulated in TNBC. We found the overexpression of MFAP2 was correlated with OS of TNBC patients and conducted Western Blot Analysis and Immunohistochemistry to further validate these findings. Moreover, we conducted GSEA and immune infiltration analysis to further explore the mechanism of MFAP2 affecting the development of TNBC and its potential to serve as an immunotherapy target. All the results demonstrated that MFAP2 might be a potential prognostic biomarker and an effective anticancer target for TNBC.

There is mounting evidence that MFAP2 promotes tumor growth [20]. For instance, MFAP2 has been demonstrated to be overexpressed in tissue from gastric carcinoma by Wang et al. Their research found that an elevated expression of MFAP2 in gastric cancer patients was strongly related to their prognosis [18]. In Zhao et al.’s study and Dong et al.’s study, MFAP2 transcription elevated in ovarian cancer and thyroid papillary cancer, respectively, and was discovered as a novel diagnostic and predictive indicator in these two categories of cancer [21, 22]. Moreover, MFAP2 levels have also been found to be elevated in head and neck squamous cell cancer according to Silveira et al.’s research [23]. The results of the studies suggest that MFAP2 may promote the development of various cancers. In addition, pan-cancer studies have also indicated that MFAP2 expression is elevated in breast cancer and may be associated with adverse prognosis in breast cancer patients [24]. However, the role of MFAP2 to serve as a biomarker to predict the adverse prognosis of TNBC patients and as an immunotherapy target has not been studied previously. In our current investigation, we have demonstrated through a combination of bioinformatics analysis and experimental validation that MFAP2 is upregulated in TNBC tissues, correlates with poor prognosis in patients, and also possesses the potential to act as an immunotherapy target. As far as we are concerned, this study firstly investigated the connection between the expression of MFAP2 and TNBC.

In our PPI network analysis, MFAP2 was found to be correlated with LOXLs (LOX, LOXL1, LOXL2 and LOXL4). The amine oxidases LOX (lysyl oxidase) and LOXL 1–4 (lysyl oxidase like-1–4) catalyze the cross-linking of elastin and collagen in connective tissue. Furthermore, these amine oxidases enhance collagen and elastin crosslinking in the tumor extracellular matrix, favoring cell motility and metastasis advancement [25]. A number of studies have demonstrated that the LOXLs affect cervical cancer, breast cancer, oral squamous cell carcinoma, and gastric cancer progression and distant metastasis [25–28]. It is likely that MFAP2 interacts with LOXLs to contribute to BC progression and metastasis.

We also found MFAP2 is related to glycolysis when conducting the GSEA analysis. Tumor cells, contrary to normal cells, tend to consume glucose via glycolysis due to increased glucose uptake and lactic acid generation. This process, is also called Warburg effect, provides significant benefits to cancer cells in tumor development, apoptosis resistance, metastasis, and immune evasion [29, 30]. Research has found that MFAP2 facilitates FOXM1 expression and regulates glycolysis in oriental cancers through the FOXM1/β-catenin signaling pathway [21]. Thus, MFAP2 may affect the prognosis of TNBC in a similar signaling pathway.

Several studies have recently found that tumor immune infiltration is linked to cancer growth. Public data mining indicated a relationship between MFAP2 transcription and TNBC immune infiltration. Th1 and Th2 helper T cells are two different subpopulations of CD4 + cells among tumor-infiltrating lymphocytes. Th2 cells are believed to release IL-4 and IL-10, which restrict the host immune system, facilitating tumors to proliferate [31, 32]. Macrophages are divided into two functional phenotypes based on tumor microenvironment signals: classically activated macrophages (M1) and alternately activated macrophages (M2). M2 has anti-inflammatory and carcinogenic properties compared to M1, which has anti-tumor properties [33]. Our research investigated MFAP2 expression is positively correlated to macrophages in TNBC and previous study has reported the overexpression of MFAP2 is positively related to markers of Th2 cells and M2 macrophages in glioma [34], implying MFAP2 performs a crucial role in tumor immunity modulation. Because of this, MFAP2 may affect the immune infiltration of TNBC in similar ways and be a target for immunotherapy in the future.

Apart from immune cell infiltration, tumor mutation burden (TMB) is another possible biomarker for predicting therapy and prognosis in multiple types of cancers [35–37]. We investigated the negative relationship between MFAP2 gene transcription and TMB in TNBC patients in our study, which suggested that MFAP2 may serve as a biomarker for PD-1/PD-L1 immunotherapy. To confirm our hypothesis, in TNBC tissues, we studied the connection between MFAP2 and PD-L1 through GSE167213 and GSE137356 datasets and employed immunohistochemistry analysis to further validate the outcome in clinical TNBC samples. Finally, we came up with a compelling result that TNBC patients with reduced MFAP2 expression had greater PD-L1 expression, which suggested we can choose appropriate immunotherapy methods according to the expression of MFAP2.

TP53 mutations can protect tumor cells from various stress stimuli, including DNA damage, oxidative stress, interactions within the tumor microenvironment, and immune system, thereby promoting cancer cell survival and tumor progression [19]. In our study, we investigated that there was a difference in MFAP2 expression level between BC patients with and without TP53 mutation. According to the literature mentioned above, here is evidence demonstrating that extracellular matrix (ECM) can influence the physical properties of the microenvironment and subsequently affect the stability of TP53 mutations. And TP53 mutation also may occur in BC progression. Besides, MFAP2 is an important component of ECM [38], we made the hypothesis that MFAP2 may be engaged in breast cancer tumorigenesis through affecting the stability of TP53 mutation. Moreover, TP53, transcription factor, can regulate the expression of various proteins to establish a pro-oncogenic tumor microenvironment. This reminds us that MFAP2 may be regulated by TP53 directly or indirectly, thereby promoting the generation of the tumor microenvironment. In conclusion, the specific mechanisms underlying the interaction between MFAP2 and TP53 mutations remain unclear and require further investigation in future studies. This provides us with a potential direction for further in-depth research on MFAP2 in the future.

In our study, we demonstrated the overexpression of MFAP2 in TNBC tissues and found that the expression of MFAP2 was linked to a poorer prognosis. However, limitations still exist in our study. At present, the mechanism of MFAP2 affecting the development of TNBC remains to be explored. Further research into the mechanism by which MFAP2 affects the TNBC is needed to provide insight into TNBC progression. Moreover, more animal model tests and clinical experiments are still needed to validate MFAP2 functions.

## Conclusion

MFAP2 plays an important role in the progression of TNBC and may be a promising prognostic biomarker as well as a feasible target for immunotherapy in TNBC.

### Supplementary Information

Supplementary file 1 (CSV 23 KB)

Supplementary file 2 (CSV 20 KB)

Supplementary file 3 (CSV 31 KB)

Supplementary file 4 (CSV 27 KB)

Supplementary file 5 (CSV 1 KB)

## Data Availability

The data support the findings of this study are incorporated into the manuscript and available from the corresponding author upon reasonable request.
